# Effects of H_2_O_2_ Treatment Combined With PI3K Inhibitor and MEK Inhibitor in AGS Cells: Oxidative Stress Outcomes in a Model of Gastric Cancer

**DOI:** 10.3389/fonc.2022.860760

**Published:** 2022-03-16

**Authors:** Luca Savino, Maria Carmela Di Marcantonio, Carmelo Moscatello, Roberto Cotellese, Lucia Centurione, Raffaella Muraro, Gitana Maria Aceto, Gabriella Mincione

**Affiliations:** ^1^ Department of Innovative Technologies in Medicine and Dentistry, University ‘G. d’Annunzio’ of Chieti–Pescara, Chieti, Italy; ^2^ Department of Medical, Oral and Biotechnological Sciences, University ‘G. d’Annunzio’ of Chieti–Pescara, Chieti, Italy; ^3^ Department of Medicine and Aging Sciences, University ‘G. d’Annunzio’ of Chieti–Pescara, Chieti, Italy

**Keywords:** gastric cancer, ErbB receptors, reactive oxygen species, base excision repair system, PI3K and MAPK inhibitors

## Abstract

Gastric cancer is worldwide the fifth and third cancer for incidence and mortality, respectively. Stomach wall is daily exposed to oxidative stress and BER system has a key role in the defense from oxidation-induced DNA damage, whilst ErbB receptors have important roles in the pathogenesis of cancer. We used AGS cells as an aggressive gastric carcinoma cell model, treated with H_2_O_2_ alone or combined with ErbB signaling pathway inhibitors, to evaluate the effects of oxidative stress in gastric cancer, focusing on the modulation of ErbB signaling pathways and their eventual cross-talk with BER system. We showed that treatment with H_2_O_2_ combined with PI3K/AKT and MEK inhibitors influenced cell morphology and resulted in a reduction of cancer cell viability. Migration ability was reduced after H_2_O_2_ treatment alone or combined with MEK inhibitor and after PI3K/AKT inhibitor alone. Western blotting analysis showed that oxidative stress stimulated EGFR pathway favoring the MAPKs activation at the expense of PI3K/AKT pathway. Gene expression analysis by RT-qPCR showed *ErbB2* and *OGG1* increase under oxidative stress conditions. Therefore, we suggest that in AGS cells a pro-oxidant treatment can reduce gastric cancer cell growth and migration *via* a different modulation of PI3K and MAPKs pathways. Moreover, the observed *ErbB2* and *OGG1* induction is a cellular response to protect the cells from H_2_O_2_-induced cell death. In conclusion, to tailor specific combinations of therapies and to decide which strategy to use, administration of a chemotherapy that increases intracellular ROS to toxic levels, might not only be dependent on the tumor type, but also on the molecular targeting therapy used.

## Introduction

Gastric cancer (GC) is an important public health problem worldwide due to its high mortality. Currently, GC is the fifth most frequently diagnosed cancer and it is the third most lethal cancer worldwide (1–3).

This high mortality rate is associated with an absence of significant symptoms in the early stages, and a lack of validated screening programs and cancer healthcare in developing countries. As a result, many cases of GC are diagnosed at an advanced stage and thus have poor prognosis.

GC is a complex and multifactorial disease with many inherited and environmental factors involved in its onset, including the genetic characteristics of the host, infectious agents [such as *Helicobacter pylori* (4), Epstein Barr virus], and dietary habits (5). Depending on tumor characteristics and stage (6), the current treatment modalities include combinations of surgery, chemotherapy/targeted therapy, and radiation therapy (7–9). Moreover, several recent targeted therapy trials have been unsuccessful (10, 11) and, to date, standard treatments of advanced GC consist of 5-FU/platinum based chemotherapy and only two biological agents, trastuzumab for HER2 positive tumors and ramucirumab for chemo-refractory patients (12, 13). Therefore, even with maximal modality therapies, prognosis for gastric cancer remains poor, with 5-year survival rates of 25% to 35% for loco-regional disease (8, 14, 15), and median survival for advanced disease of 10 months to 14 months (16, 17).

Receptor tyrosine kinase signaling has also been shown to have roles in gastric cancer and in particular tyrosine kinases receptors of ErbB family (18–20). The ErbB family of proteins comprises four receptor tyrosine kinases: EGFR (ErbB1/HER1), ErbB2 (HER2/neu), ErbB3 (HER3), and ErbB4 (HER4). With respect to the ErbB receptors ligands, around two dozen have been described for mammals (21–23). When these receptors are activated by ligand binding, they can form homodimers or heterodimers, which is followed by activation of phosphorylation cascades that defines the signal that is transduced inside the cell (24). In particular, this leads to activation of the “Akt-mammalian target of rapamycin” (mTOR) PI3K and Ras–Raf mitogen-activated protein kinase (MAPK)/extracellular signal-related kinase (ERK) pathway that plays an important role in mediating cell survival and cell proliferation, respectively (25). The EGFR has been reported to be overexpressed in 27%−64% of gastric tumors (26). It has also been suggested that high gene amplification of the EGFR is related to poor patient outcome. However, a 2013 meta-analysis that compared five studies that included 1,600 patients reported that in GC EGFR expression was not an independent predictor of survival (27). ErbB2 amplification/overexpression has been reported for 6–30% of GCs, with this variability attributable in part to histological subtypes and primary tumor location (28). In a comparison with diffuse-type tumors, ErbB2 overexpression showed greater prevalence for intestinal-type and gastroesophageal junction tumors (29, 30). Many studies have shown ErbB2 positivity to be indicative of poor patient prognosis; ErbB2 expression and gene amplification are used as a biomarker for targeted therapy of patients with GC (27). The monoclonal antibody trastuzumab binds to the ErbB2 extracellular domain, through which it blocks ErbB2 receptor cleavage, inhibits its dimerization, and induces antibody-dependent cellular cytotoxicity. In the phase III ToGA trial, the combination of trastuzumab with standard cisplatin and 5-fluorouracil therapies improved overall survival from 11.1 to 13.8 months for patients with ErbB2-amplified gastric adenocarcinomas (31). ErbB3 and ErbB4 can be mutated in GC, although at low frequency (<10%) (32, 33). Nielsen et al. demonstrated down-regulation of both ErbB4 and NRG4, its specific ligand, in cancer tissues (34).

The phosphatidylinositol 3-kinase (PI3K)-AKT and MAPK kinase (MEK)–extracellular-related kinase (ERK) pathways play a central role in transmitting the oncogenic signals downstream of ErbB RTKs thus contributing to cancer phenotype in terms of cell cycle progression, survival, metastasis, reprogramming of metabolism and resistance to chemotherapy. GC harbors some of the highest rates of oncogenic alterations in PI3K-AKT but the efforts to translate knowledge of these genetic alterations into clinical practice have encountered limited clinical success. Thus, identification and refinement of molecular mechanisms involved in gastric cancer in response to therapeutic agents will allow to define the rationale for the best therapeutic approach targeting the PI3K pathway. Therefore, since inhibition of PI3K-AKT and MEK-ERK signaling can diminish cell growth and promote cell death, targeting these signaling pathways by LY294002 and PD98059, inhibitors of PI3K-AKT and MEK-ERK signaling respectively, is being evaluated in clinical trials for cancer therapeutics. Yao J. et al. demonstrated that γ-secretase inhibition combined with PD98059 enhances cell death in GC cells partly through downregulation of WNT/β-catenin pathways (35). Another study by Qian C et al. demonstrated that in GC cell SGC7901 inactivation of ERK1/2 using PD98059 markedly enhanced JAK2 shRNA-induced cell proliferation inhibition, cell cycle arrest and apoptosis *in vitro* and in nude mice (ERK1/2 inhibition enhances apoptosis induced by JAK2 silencing in human gastric cancer SGC7901 cells (36). The efficacy of PI3K pathway inhibitor has been demonstrated by Sun L. et al. (37) in GC cell line SGC7901, where the enhanced proliferation and migration ability induced by the glycolytic enzyme alpha-enolase (ENO1) overexpression was impaired after incubation with PI3K inhibitor LY294002. Recently, the results obtained by Cai J. et al. (38), showed the ability of the poly(lactic acid/glycolic) (PLGA) nanoparticles loaded with Docetaxel and LY294002 to markedly reduced proliferative capacity and an elevated apoptosis rate *in vitro* and anti-cancer effects in an *in vivo* orthotopic GC mouse model and xenograft mouse model.

Physiologically, reactive radical-type oxygen species lead to the final formation of hydrogen peroxide (H_2_O_2_), a highly diffusible molecule (39). This molecule produced by healthy cells is involved in multiple intracellular stress response signals (40). However, when H_2_O_2_ is over-produced or inadequately detoxified, it can induce excessive intracellular production of 7,8-dihydro-8-oxoguanine (8-oxoG) (41). Base excision repair (BER) system plays a crucial role in the correction of DNA errors from guanine oxidation and then may be considered a cell protective factor (42). 8-oxoguanine DNA glycosylase (OGG1) is one of the most important enzymes of the BER system, as it is responsible for excision of 8-oxoG, one of the major oxidized bases in DNA that is highly mutagenic because it can pair with adenine as well as cytosine. Mutations due to 8-OxoG accumulate in both nuclear and mitochondrial DNA and are believed to be a major cause of cancers (43).

The most significant effects of oxidants on signaling pathways have been observed for the mitogen-activated protein (MAP) kinase/AP-1 and NF-κB pathways (44). In this scenario, ROS are involved in cell proliferation promoted by ligand-independent transactivation of receptor tyrosine kinases, with decreased receptor tyrosine kinase activation threshold and increased MAPK activation, as well as in promoting tissue invasion and metastatic dissemination (45, 46) (**Figure S1**).

Moreover, a link between EGFR signaling and DNA repair mechanisms has been shown previously (47, 48). Staffolani and colleagues demonstrated that in a human lung cell line, EGFR down-regulates wood-dust-induced OGG1 expression *via* AKT (49). However, to date, correlation between the BER and ErbB systems in gastric cancer has not been investigated. As cancer cells produce higher levels of ROS than normal cells (50), the use of drugs that can further increase ROS production to toxic levels might represent a therapeutic strategy to kill cancer cells (51). Many anticancer drugs are at present used for cancer treatments based on their activation of ROS-induced cell-death pathways, through increasing ROS production (52, 53).

Thus, we have attempted to create conditions of short times, high doses of H_2_O_2_ and use this to investigate, at multiple levels, the alterations of cellular functions. In particular, to understand the biological effects of short and severe oxidative treatment and to highlight potential interactions, we analyzed here the expression of the components of the BER and ErbB systems in AGS cells. This gastric cancer cell line serves as a model of untreated human stomach adenocarcinoma that retains the same cytological characteristics as the original malignant cells of the patient (54). Moreover, the effects of this oxidant treatment were investigated in combination with gefitinib, an EGFR inhibitor, and with specific targeting agents directed against the ErbB downstream signaling pathways, including PD98059 (MEK1 inhibitor) and LY294002 (PI3K inhibitor).

Therefore, the aim of the present study was to investigate the biological effects of short and severe oxidative stress on a model of advanced gastric tumor. Oxidative stress has been promoted *in vitro* by H_2_O_2_ treatment of human cancer cells, to mimic strong oxidative stress *in vivo* (55). Thus, the effects of oxidant treatments alone and in combination with specific target therapies directed against ErbB downstream signaling pathways were investigated to evaluate their potential combined benefits in the treatment of gastric cancer, with parallel analysis of the expression of the components of the BER system, to highlight potential interactions.

## Materials and Methods

### Cell Lines and Treatments

Primary human gastric epithelial cells would closely represent the gastric epithelium; however, to date, no primary gastric epithelial cell culture systems have been established that enable effective research on gastric diseases and disorders *in vitro*. Thus, we chose to use a human gastric adenocarcinoma cell line (AGS cells; European Collection of Authenticated Cell Cultures 89090402). These cells were derived from an untreated human adenocarcinoma of the stomach that retained the same cytological characteristics of the malignant cells obtained from the caucasian patient (50). The AGS cells were obtained from Cell Lines Services (Epplheim, Germany), and were cultured in Dulbecco’s modified Eagle’s medium (DMEM) supplemented with 4.5 g/L glucose, 2 mM L-glutamine and 10% fetal bovine serum (FBS) (EuroClone S.p.A., Pero, MI, Italy). For the experiments, these AGS cells were cultured in DMEM starvation media overnight (i.e., 0.2% FBS). Under the acute treatment conditions, the cells were pre-treated (as required) with the MEK1 inhibitor PD98059 (50 µM) and/or the PI3K inhibitor LY294002 (25 µM) (Cell Signaling Technology, Beverly, MA, USA) for 2 h, and then stimulated with human recombinant EGF (50 ng/mL) and/or H_2_O_2_ (10 mM) (Sigma Aldrich, St. Louis, MO, USA). For the chronic treatments, these were added together (as required) for 24 h or 48 h, with 50 µM H_2_O_2_. The AGS cells were also treated with 1 μM gefitinib (as required) for 1 h, which corresponds to the serum concentration reported in clinical trials. Stock solutions of the EGFR kinase inhibitor gefitinib (ZD1839; AstraZeneca, Macclesfield, UK) were prepared in dimethylsulfoxide and stored at -20°C.

### Cell Morphology

The AGS cells were seeded on glass coverslips, and then serum starved in DMEM with 0.2% FBS overnight. Following the required pre-treatments (PD98059, LY294002, gefitinib; see above), 10 mM H_2_O_2_ was added for 15 min. The cells were then fixed with 70% ethanol and stained with 1% toluidine blue solution (TAAB Laboratory Equipment Ltd, UK). Digital images from three different experiments were obtained under light microscopy (Axioskop 40; Carl Zeiss) equipped with a videocamera (Coolsnap; Photometrics), using the MetaMorph 6.1 software system (Universal Imaging Corp, Molecular Device Corp, CA, USA).

### Cell Proliferation Assay

Cell proliferation was determined using the 3-(4,5-dimethylthiazol-2-yl)-5-(3-carboxymethoxyphenyl)-2-(4-sulfophenyl)-2H-tetrazolium (MTS) assay (Promega, Madison, WI, USA). The cells were seeded in 96-well plates, serum-starved overnight, and then treated as required, with 50 µM H_2_O_2_, 50 ng/mL EGF, 50 µM PD98059, 25 µM LY294002 and/or 1 µM gefitinib, with individual or combined treatments for 24 h. The day after, MTS solution (as 10% total medium volume) was added to each well, and following incubation for 1 h at 37°C, the cell viabilities were determined by colorimetric absorbance at 490 nm, using a microplate reader (Glomax, Multi Detection System, Promega).

### Wound Healing Migration Assay

The AGS cells were seeded into 12-well plates, grown to 100% confluence, and then wounded with a sterile pipette tip, to remove cells using linear scratches. Then, the cells were washed with phosphate-buffered saline, and serum-free medium was added, containing (as required) 50 μM H_2_O_2_, 50 ng/mL EGF, 50 µM PD98059, 25 µM LY294002, and/or 1 μM gefitinib, as individual or combined treatments. The progress of cell migration near the crossing point was photographed immediately after injury (time 0) and after 24 h and 48 h. The wound healing was calculated by measuring the width of the scratch along the border using the Image J64 analysis software, with quantification according to the following: change in wound closure (Δ) = (wound width, time 0) – (wound width, time 24/48 h); i.e., the decrease in the gap width from time 0. This defined higher Δ values as greater cell migration, where the cells had migrated more into the space left after the wounding; thus, a score of zero would indicate no wound closure from the control.

### Total RNA Isolation and Real-Time Quantitative PCR Analysis

Total RNA was extracted from the AGS cells following the required treatments using Trizol (Life Technologies, Carlsbad, CA, USA), according to the manufacturer instructions. RNA samples were assessed for purity and quantified using a spectrophotometer (Nanodrop 1000; Thermo Fisher Scientific, Waltham, MA, USA), with reverse transcription performed using GoTaq 2-Step qRT-PCR kits (Promega), according to the manufacturer instructions. The levels of BER and of ErbB gene expression were investigated using SYBR green quantitative real-time PCR (qRT-PCR) analysis (StepOne 2.0; Applied Biosystems, Carlsbad, CA, USA). The data were analyzed by the comparative Ct method and are given as 2^−ΔΔCt^ + standard deviation (SD). As required with this method, the mRNA levels of the target genes were normalized by the ratio between the mean values for the endogenous housekeeping gene (β-glucuronidase; GUSB) in each sample versus quiescent cells.

### Protein Extraction and Western Blotting

The AGS cells were serum-starved overnight in DMEM with 0.2% FBS and treated as required (see above). The plates were then washed with ice-cold Ca^2+/^Mg^2+^-free phosphate-buffered saline and lysed in freshly prepared lysis buffer (2 mM Na_3_VO_4_, 4 mM sodium pyrophosphate, 10 mM sodium fluoride, 50 mM HEPES pH 7.9, 100 mM NaCl, 10 mM EDTA, 1% Triton X-100, 2 µg/mL leupeptin, 2 µg/mL aprotinin, 1 mM phenylmethylsulfonyl fluoride). Protein concentrations were determined using the BCA protein assay (Thermo Fisher Scientific). The cell extracts were subjected to 4% to 20% sodium dodecyl sulfate–polyacrylamide gel electrophoresis (pre-cast gels; Bio-Rad Laboratories, Hercules, CA, USA), with the separated proteins transferred to polyvinylidene difluoride membranes. After blocking the nonspecific binding sites with albumin or nonfat dry milk, the membranes were incubated with antibodies against the following proteins (as required): phospho-p44/42 MAPK, phospho-Akt, and phospho-EGFR (Tyr1068) (Cell Signaling Technology); EGFR (Santa Cruz Biotechnology, Santa Cruz, CA, USA); β-actin (Sigma-Aldrich) (protein loading control). Secondary antibodies were HRP-conjugated anti-rabbit or anti-mouse (Bethyl Laboratories, Montgomery, TX, USA), and the immune complexes were visualized using the ECL Western blotting detection system (Euroclone).

### Statistical Analysis

The data are reported as the representative values of three independent experiments. Data are expressed as the means + SD and analyzed by unpaired t test (two-tailed P value) and one-way analysis of variance (ANOVA) followed by Bonferroni *post-hoc* test. Statistical significance was accepted at p <0.05. As regards gene expression analysis, the comparative 2^−ΔΔCt^ method was used to quantify the relative abundance of mRNA and then to determine the relative changes in individual gene expression (relative quantification). All analyses were performed using SPSS software. The gene expression data obtained from this study were clusterized with the Multiexperiment viewer v4.0 (MeV4.0) program (56), determining the molecular relationships among analyzed genes, without taking into account their relative pathways.

## Results

### Effects of Oxidative Stress on AGS Cell Morphology

**Figure 1:**
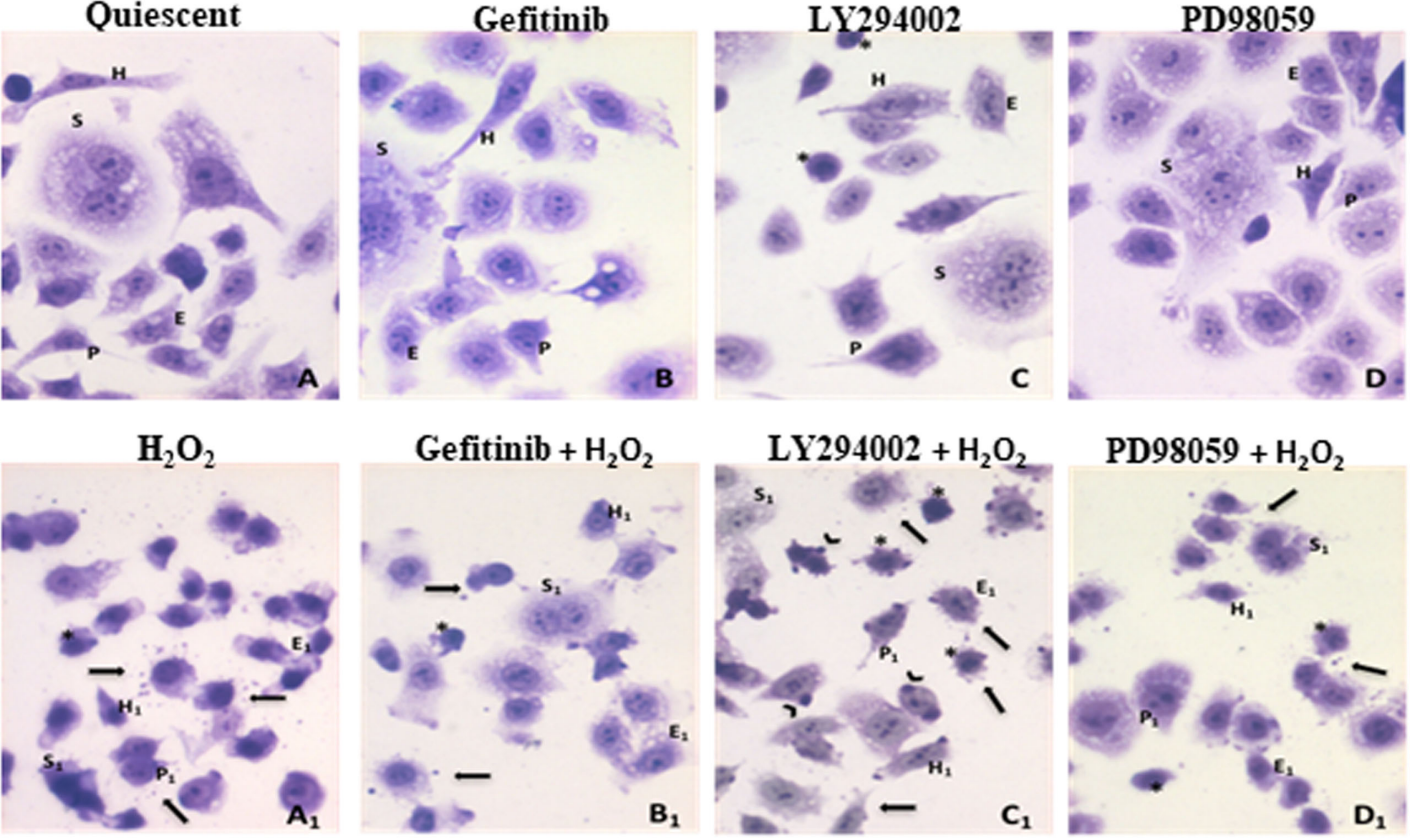
Effects of oxidative stress induced by H_2_O_2_ treatments combined to ErbB pathway inhibitors on AGS cells. **(A)**-Quiescent cells. Different morphological cellular phenotypes: (H) mononucleated and elongated cells, “hummingbird”-like; (S) multinuclear large syncytial cells; (E) epithelial-like polygonal little cells; (P) cells exhibiting a very thin prolongation from the cellular body. **(B)**-Gefitinib treatment (1µM), **(C)**-LY294002 treatment (25µM), cells with heterochromatic nucleus were evidenced (asterisks), **(D)**-PD98059 treatment (50µM). **(A1–D1)** Inhibitors and H_2_O_2_ (10mM) co-exposure: after H_2_O_2_ exposure, in all cases, a general shrinkage of all different cell phenotypes was observed (H_1_, S_1_, E_1_, P_1_ represent the original features still recognizable), in addition to the presence of extracellular vesicles (arrows) and many small, round cells with heterochromatic nuclei (asterisks). **(C1)** Larger and darker vesicles emerging from cell surface are detected after LY294002 and H_2_O_2_ exposure (arrows). Toluidine blue staining (40X).

The effects of H_2_O_2_ treatment on the AGS cell morphology were analyzed in quiescent and pre-treated cells grown in serum-free medium, to avoid interference from exogenous growth factors. Besides the expected AGS cells organized in clusters of numerous polygon-shaped little cells (E) (57), different main morphological cell phenotypes showing euchromatic nuclei with numerous prominent nucleoli are identified: mononucleated and elongated cells, “hammingbird”-like (H), resembling hummingbird phenotype (H), characteristic of the *H. pylori*-induced stress (58); syncytial giant cells with multiple nuclei (S); some cells with short prolongments from the cellular body (P) (**Figure 1A**). After the different pre-treatments (i.e., gefitinib, LY294002, PD98059; **Figures 1B–D**), there were fewer cells classified as epithelial-like, due to more rounded shaped cells, which were mainly visible after the PD98059 treatment. Furthermore, after treatment with LY294002, there were also numerous small round cells with a heterochromatic nucleus (**Figures 1B–D**). Following the acute H_2_O_2_ exposure the cell morphologies showed a general shrinkage for all of these phenotypes (**Figures 1A1–D1**). Interestingly, under these oxidative stress conditions, perimembranous release of extracellular vesicles (EV) was seen. Sometimes larger and darker vesicles emerged from cell surface. These vesicles were more numerous in combination with the LY294002 pre-treatment (**Figure 1C1**), which could not be considered as apoptotic micronuclei, as they were negative to DAPI counterstaining (data not shown).

### Effects of Oxidative Stress on Viable Cell Proliferation

The cellular response to exposure to either acute (single high dose for short times) or chronic (long term at low doses) oxidizing agents is different. Acute exposure to oxidative stress triggers a series of intracellular defense mechanisms that counteract the damage. The cells will die by apoptosis or necrosis, if these defence processes are insufficient. However, if the cells survive, exposure also increases many antioxidant defenses. Thus, in cells chronically exposed to sublethal stress, a series of adaptive responses occur that can prevent or reduce damage and death.

**Figure 2:**
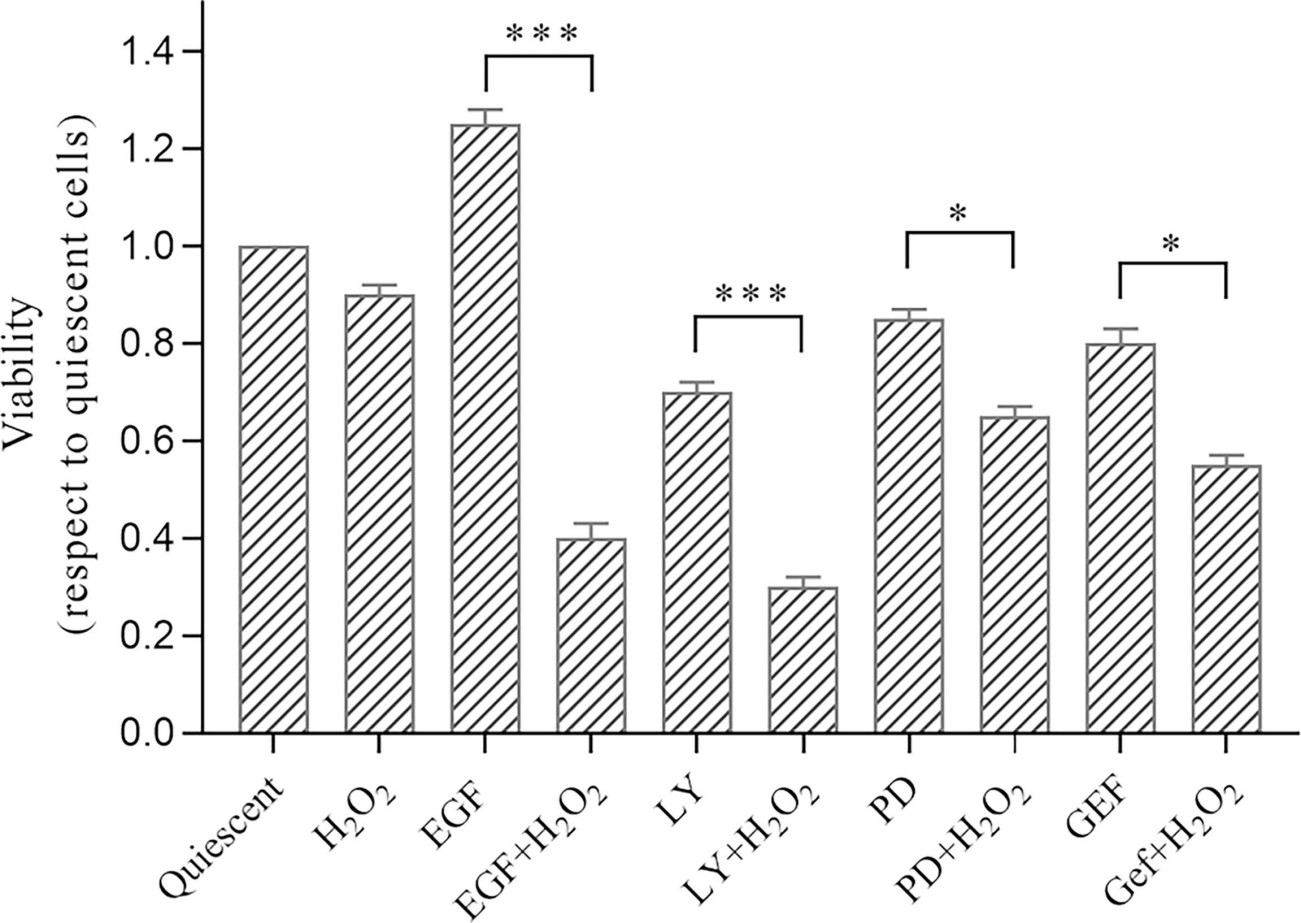
Analysis of effects of treatments with H_2_O_2_ combined with ErbB pathway inhibitors on AGS cell viability. Subconfluent cells were serum-starved overnight and treated with 50 ng/mL EGF, 1 μM gefitinib (GEF), 25 µM LY294002 (LY), 50 μM PD98059 (PD), and/or 50 μM H_2_O_2_ or untreated (Quiescent). Cell proliferation (Viability) was evaluated after 24 h (MTS assay), with the data expressed normalized to quiescent cells. Data are means ± standard deviation of four independent experiments, each in quintuplicate. *p < 0.05; ***p < 0.001 (student’s t-tests).

Thus, to evaluate the effects of oxidative stress induced by H_2_O_2_ on proliferation and migration of the AGS cells, we treated these cells for 24 h at low dose of 50 µM H_2_O_2._ For viability assay, AGS cells were seeded in 96-well plates, and semiconfluent cultures were serum-starved (0.2%) overnight. The cells were then treated as control (quiescent) cells or with 50 ng/mL EGF, 25 µM LY294002, 50 µM PD98059, or 1 µM gefitinib, without and with 50 µM H_2_O_2_ for chronic treatment for 24 h. The mitogenic effects of the treatments were determined using the MTS assay (**Figure 2**). After 24 h of EGF treatment, there was increased cell proliferation compared to the untreated (quiescent) cells, as aspected, due to the well-known mitogenic effects of EGF on epithelial cells pathway (59).

Interestingly, a reduction in cell proliferation was seen for LY294002 treatment, which is in accordance with the well-established effect on cell survival and proliferation mediated through the PI3K/AKT pathway (60).

Here, the H_2_O_2_ treatment alone did not induce significant proliferative changes compared to the untreated cells. However, the H_2_O_2_ treatment resulted in significant reduction in cell proliferation when in synergy with LY294002 (alone, -30.0%; +H_2_O_2_, -70.0%; p <0.001), PD98059 (alone, -15.0%; +H_2_O_2_, -35.0%; p <0.05), and gefitinib (alone, -20.0%; +H_2_O_2_, -45.0%; p <0.05). Of note, the well-known proliferative effects of EGF were dramatically reduced by the H_2_O_2_ treatment (EGF alone, +25.0%; +H_2_O_2_, -60.0%; p <0.001).

### Effects of Oxidative Stress on Cell Migration

**Figure 3:**
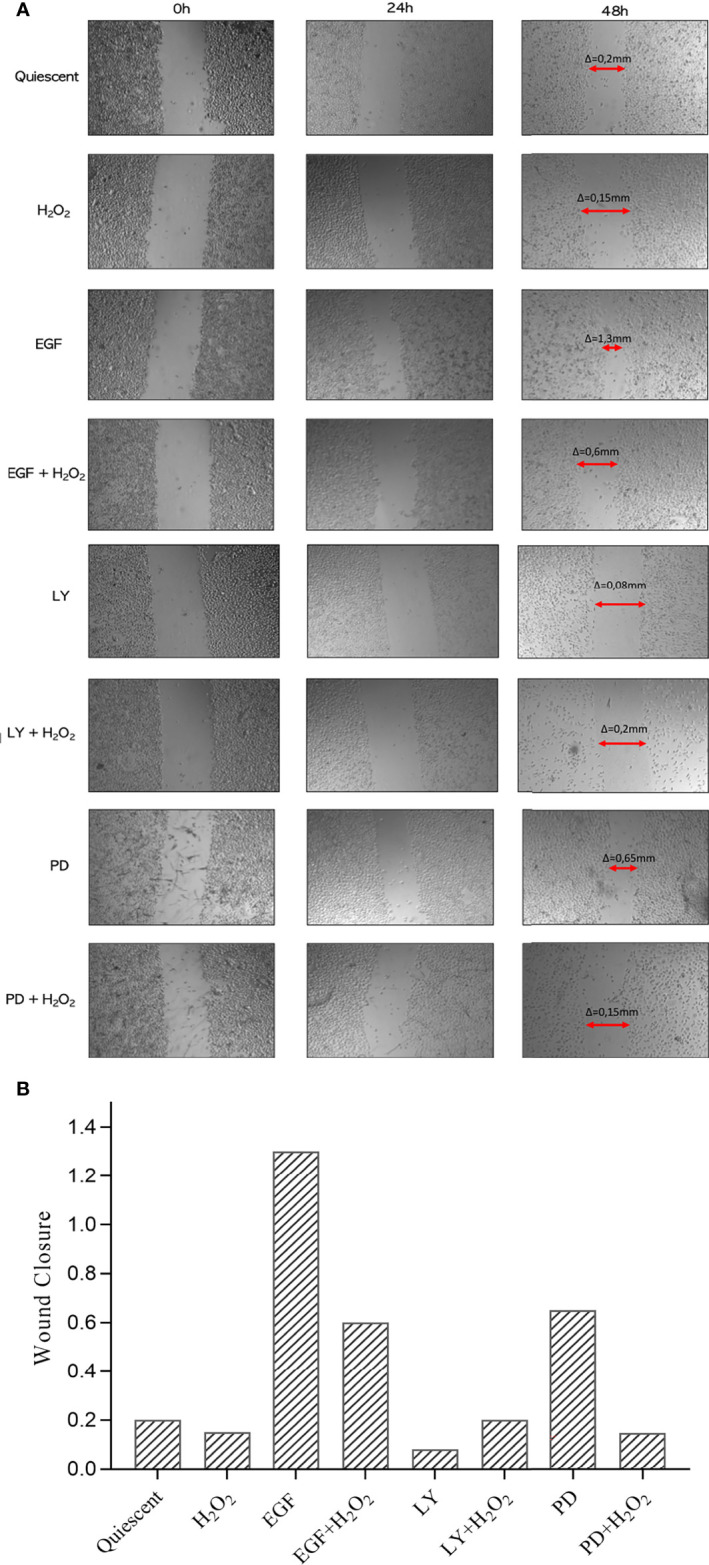
(Effect of treatments on migratory behavior of AGS cells. **(A)** Wound Healing assay was performed in confluent monolayers and the area colonised by the cells estimated after untreated (Quiescent) and treated with 50 ng/mL EGF, 25 μM LY294002 (LY), 50 μM PD98059 (PD), and/or 50 μM H_2_O_2_, at time 0 and after 24 h and 48 h Arrows, closure of wound compared to time 0 (Δ = gap width at 48 h minus that at time 0). **(B)** Percentages of wound clousure at 48 h under each conditions as in **(A)** are plotted. Representative of four independent experiments. PD: PD98059; LY: LY294002.

As tumor cell migration has an important role in the development of metastatic disease, a classic wound healing assay was used to quantify the migration of the AGS cells into the scratched area (i.e., wound) without and with low dose of 50 µM H_2_O_2_, for 24 h and 48 h alone or combined with the ErbB system inhibitors, as compared to the control cells. This cell migration into the wound gap was monitored at time 0 and after 24 h and 48 h (**Figure 3**). Reduced cell migration was seen for the H_2_O_2_ treatment alone, with slightly less wound closure at 48 h compared to the untreated cells [Δ (decrease in gap width from time 0) = 0.15 vs 0.20 mm for control, untreated, cells]. In contrast, there was a greater wound closure at 48 h with the PD98059 treatment compared to the control (Δ = 0.65 vs 0.20 mm), which suggested promotion of cell migration through inhibition of the MAPK pathway. For the H_2_O_2_ plus PD98059 treatments, the cell migration (Δ = 0.15 mm) was the same of H_2_O_2_ treatment alone (Δ = 0.15 mm) but it was slightly lower than the control (Δ = 0.20 mm) and than PD98059 alone (Δ = 0.65 mm). These findings suggest that the MAPK pathway is responsible for the reduced cell migration. In contrast, the EGF treatment improved cell migration (and hence showed greater gap closure) compared to the control cells (Δ = 1.30 vs 0.20 mm), as expected (61). However, H_2_O_2_ co-treatment with EGF reduced the EGF-induced cell migration (Δ = 1.30 vs 0.60 mm with H_2_O_2_). A possible inducing role of AKT activation on cell migration was observed after the LY294002 treatment. Indeed, LY294002 treatment inhibits cell migration compared to untreated cells (Δ = 0.08 vs 0.20 mm control), which suggests a role for AKT activation in cell migration. No difference in wound healing was seen after LY294002 co-treatment with H_2_O_2_ (Δ = 0.20 mm), as compared to untreated cells. For gefitinib, no changes were seen for any of these conditions (data not shown).

### ErbB Receptors Gene and Protein Expression in AGS Cells Under Oxidative Stress

ErbBs, and in particular ErbB2, have critical roles in the regulation of cell growth. Activation of the EGFR downstream pathways leads to increased cell proliferation and migration, and its overexpression is associated with worse prognosis for various carcinomas (27). In the present study, the modulation of ErbB signaling in response to an acute single high dose of 10 mM H_2_O_2_ was investigated in the AGS gastric cancer cell line.

**Figure 4:**
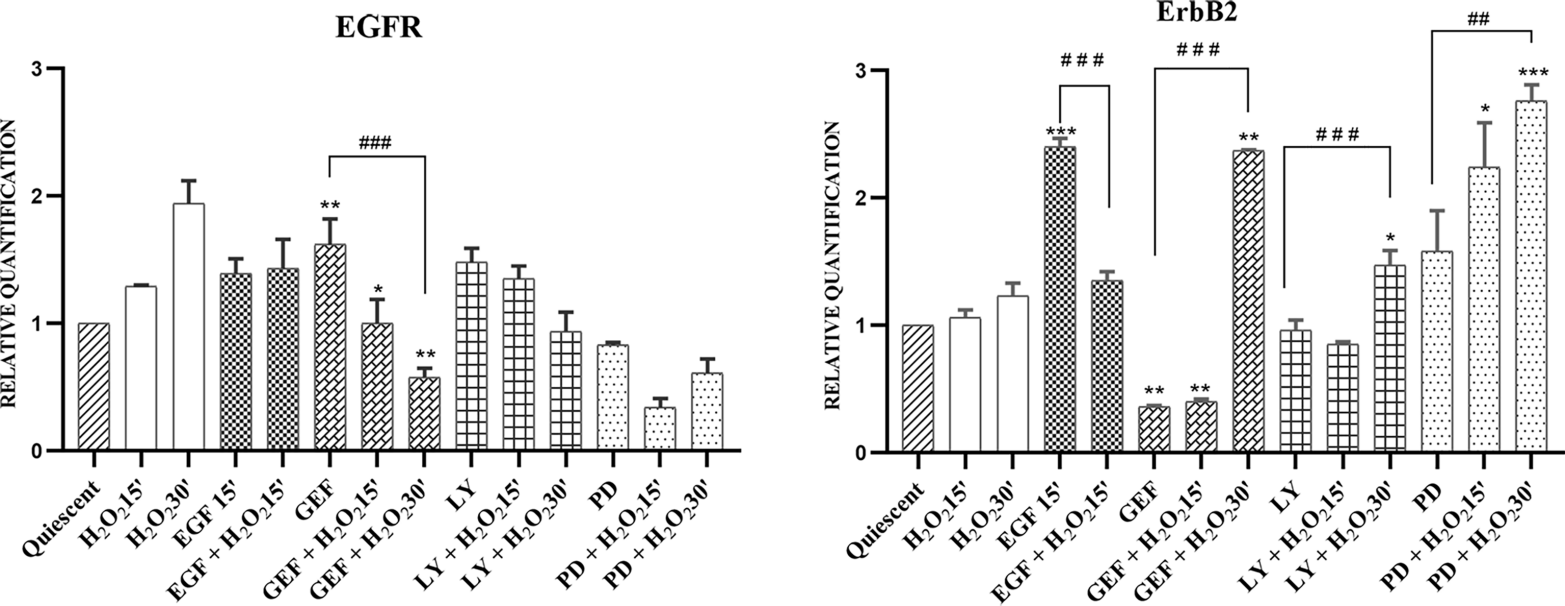
mRNA expression of EGFR and ErbB2 in AGS cells. Total RNA was extracted from serum-starved cells untreated (Quiescent) and treated with 50 ng/mL EGF, 1 μM gefitinib (GEF), 25 μM LY294002 (LY), 50 μM PD98509 (PD), and/or 10 mM H_2_O_2_, for 15 and 30 min. Analysis was carried out by RT-qPCR. As for gene expression analysis, data were calculated using the 2^−ΔΔCt^ method, normalized to GUSB (β-glucuronidase) mRNA levels, and expressed relative to control (calibrator sample, defined as 1.00). Data are expressed as means of three independent experiments. *p < 0.05; **p < 0.01; ***p < 0.001 (student’s t-tests, *vs* quiescent cells); ^##^p <0.01; ^###^p<0.001 (ANOVA followed by Bonferroni *post-hoc* test, within treatments). GEF, gefitinib; LY, LY294002; PD, PD98059.

The RT-qPCR was performed on cells treated with 50 ng/mL EGF, 25 µM LY294002, 50 µM PD98059, or 1 µM gefitinib, without and with 10 mM H_2_O_2_, at different short exposure times (15/30 min) compared with untreated cells (quiescent) as control. The expression of all of the four ErbB family members was analysed (**Figure 4**).

The gene expression results showed that H_2_O_2_ alone stimulus induced time-dependent EGFR up-regulation. Treatment with H_2_O_2_ did not alter the moderate EGF-induced increases in *EGFR* gene expression. In contrast, both gefitinib (p <0.05) and LY294002 treatments increased *EGFR* gene expression compared to the untreated cells. H_2_O_2_ co-treatments showed time-dependent down-regulation of increased *EGFR* gene expression levels. In particular, this trend resulted significant after treatment by gefitinib with H_2_O_2_ (p<0.01). Interestingly, while PD98059 treatment alone slightly reduced EGFR gene expression, the combination with H_2_O_2_ showed a greater reduction, even if transient, of the *EGFR* gene expression compared to the untreated cells (**Figure 4**). The analysis of *ERBB2* gene expression indicated that H_2_O_2_ treatment alone had no effect compared to the expression in untreated cells, while a time-dependent increase in *ERBB2* gene expression was shown in the co-treatments with gefitinib (p<0.01), LY294002 (p<0.05), and PD98059 (p<0.001). Moreover, EGF treatment up-regulated *ERBB2* gene expression compared to the untreated cells (p <0.001), an effect that was reduced by ~40% under the added H_2_O_2_ stimulus (**Table S1**). Under these experimental conditions, the expression levels of *ERBB3* and *ERBB4* showed only small, and generally not significant effects (data not shown).

**Figure 5:**
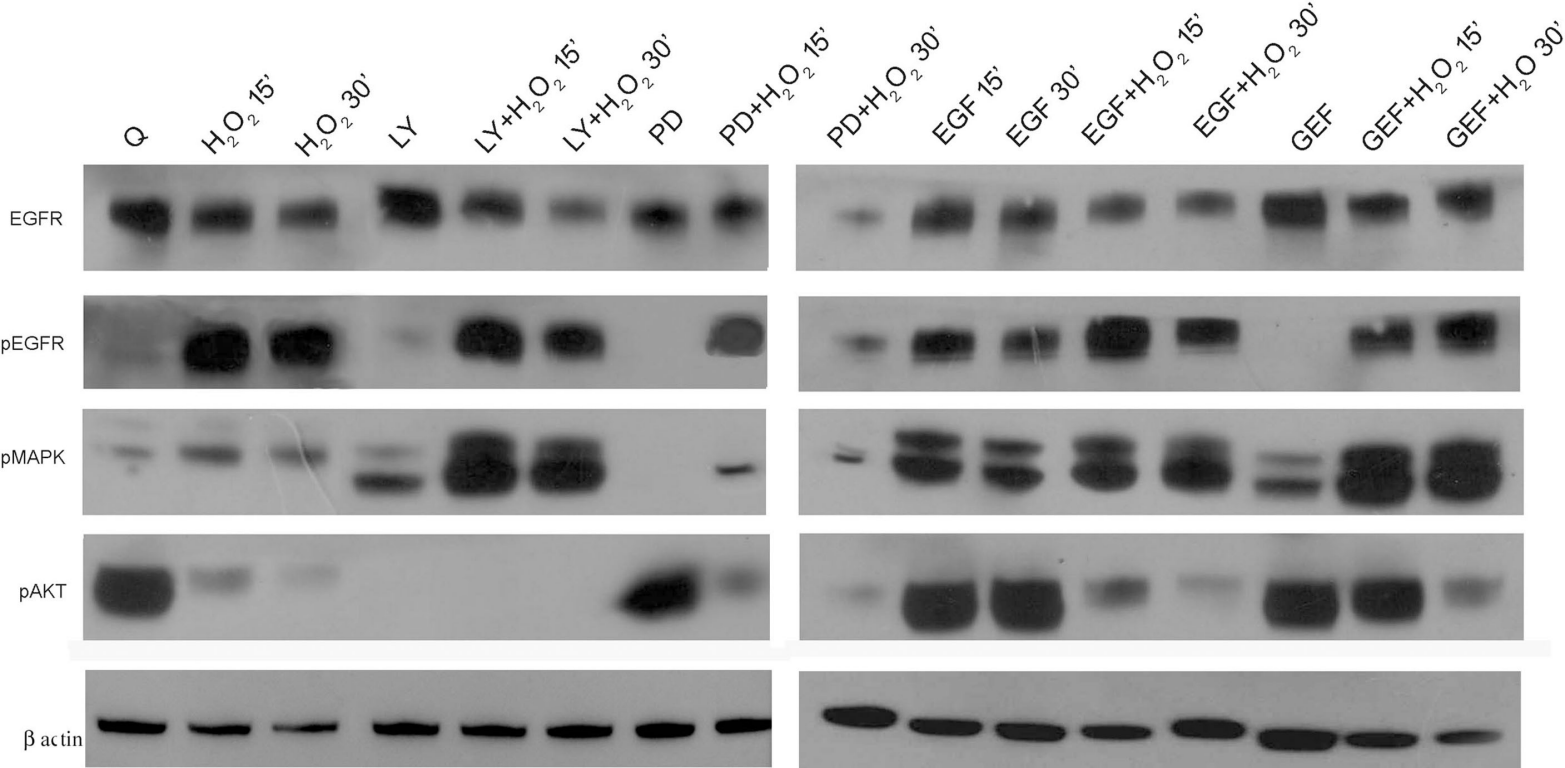
EGFR pathways protein expression analysis. Representative Western blotting of serum-starved cells untreated (Q) and treated with 50 ng/mL EGF, 1 μM gefitinib (GEF), 25 μM LY294002 (LY), 50 μM PD98059 (PD), and/or 10 mM H_2_O_2_, for 15 and 30 min. Whole cell lysates were analyzed by Western blotting for expression of total EGFR, phospho (p)EGFR, pMAPK, pAKT, and β-actin (as indicated). Data are representative of six independent experiments. Q, Quiescent; LY, LY294002; PD, PD98059; GEF, gefitinib.

Subsequently, we investigated the effects of this oxidative stress on some of the components of the EGFR downstream signaling pathway in AGS cells by Western blotting analysis (**Figure 5**). First, the levels of total and phosphorylated EGFR (pEGFR) were assessed. Interestingly, there was a significant increase in pEGFR after these acute treatments with H_2_O_2_ alone and when combined with EGF, LY294002, and PD98059, as compared to the quiescent untreated cells. Interestingly, co-treatment with H_2_O_2_ and gefitinib showed significant EGFR activation despite its known inhibiting role, which was confirmed here by the gefitinib alone treatment (**Figure 5**). EGFR phosphorylation activates downstream signaling proteins, including PI3K/AKT and p44/p42 MAPKs. Their phosphorylation-mediated activation was investigated using specific anti-phosphotyrosine antibodies. In the untreated cells, the AKT protein showed high levels of basal activation, which were significantly reduced in a time-dependent manner by the H_2_O_2_ treatment. The control AKT activation was also virtually abolished by the LY294002 treatment, both alone and in combination with H_2_O_2_, while for EGF, gefitinib, and PD98059, it was decreased with the combined H_2_O_2_ treatments. Interestingly, activation of the MAPKs proteins was increased under all these treatments by the addition of H_2_O_2_, although to different extents. Of note, the co-treatment with EGF and H_2_O_2_ preserved the activated status of the MAPK pathway, while it reduced activated AKT.

### Base Excision Repair Gene Expression Under Oxidative Stress

The production of ROS, such as H_2_O_2_, superoxide and hydroxyl radicals, has been linked to tumor initiation and progression (62). The OGG1 and MutY homolog (MUTYH) glycosylases constitute the 8-oxoG repair pathway and, in association with other molecules such as APE-1 and JUN/AP1, they cooperate in the repair of DNA damage induced by oxidative stress. In gastric cancer, there are elevated levels of the 8-oxoG mutagenic base, and some of the DNA repair enzymes are specific for this substrate when it is incorrectly incorporated into DNA, such as the OGG1 BER pathway.

**Figure 6:**
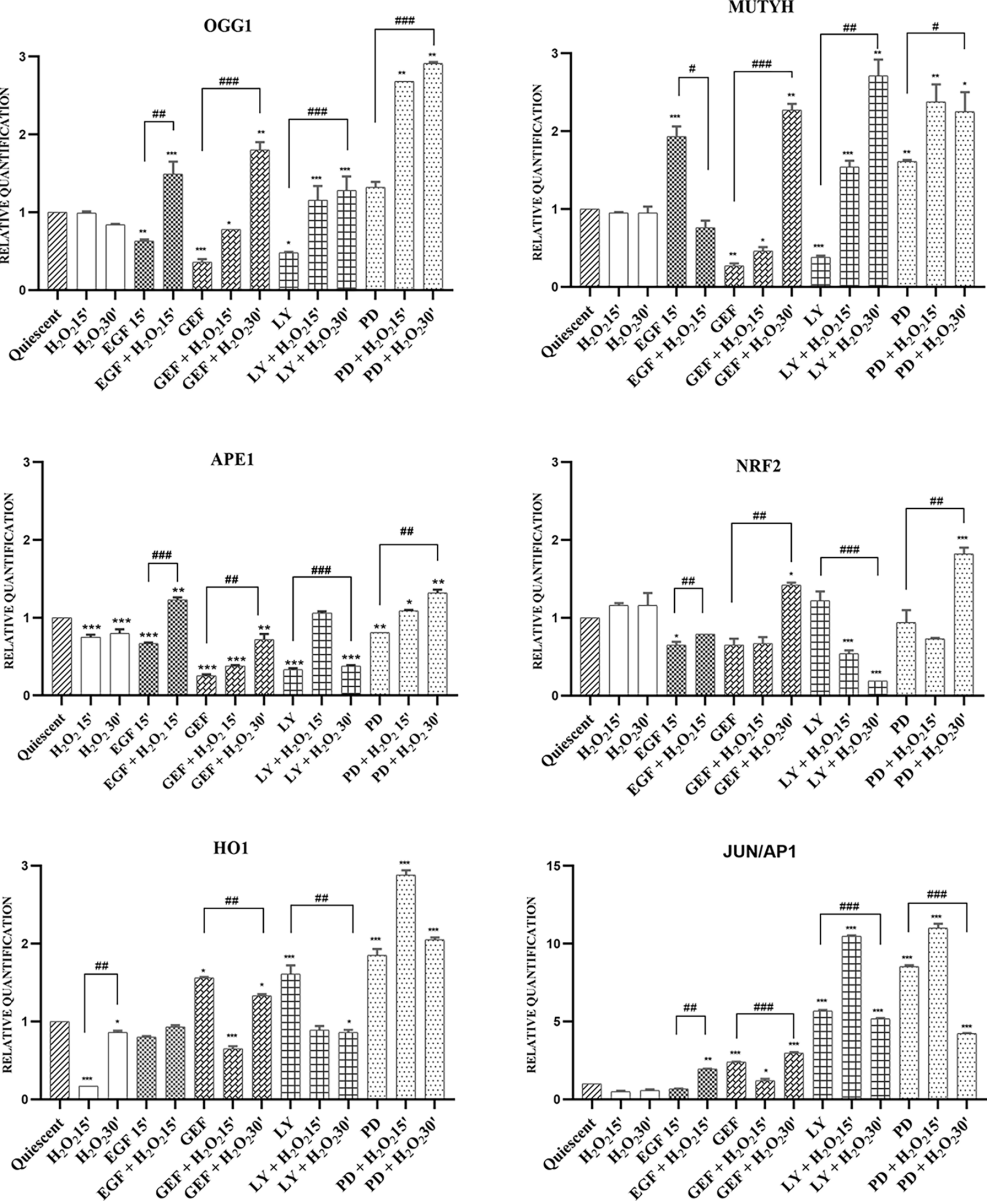
mRNA expression of BER system in AGS cells. Total RNA was extracted from serum-starved cells untreated (Quiescent) and treated with 50 ng/mL EGF, 1 μM gefitinib (GEF), 25 μM LY294002 (LY), 50 μM PD98059 (PD), and/or 10 mM H_2_O_2_, for 15 and 30 min. Analysis was carried out by RT-qPCR, for mRNA expression of the *OGG1*, *MUTYH*, *APE1*, *NRF2*, *HO1* and *JUN/AP1* genes (as indicated). As for gene expression analysis, data were calculated using the 2^−ΔΔCt^ method, normalized to GUSB (β-glucuronidase) mRNA levels, and expressed relative to control (calibrator sample, defined as 1.00). Data are expressed as means of three independent experiments. *p < 0.05; **p < 0.01; ***p < 0.001 (student’s t-tests, *vs* quiescent cells); ^#^p < 0.05; ^##^p < 0.01; ^###^p < 0.001 (ANOVA followed by Bonferroni *post-hoc* test, within treatments). GEF, gefitinib; LY, LY294002; PD, PD98059.

In response to an acute single high dose of 10mM H_2_O_2,_ we investigated the modulation of the leading actors of BER, as the *OGG1*, *MUTYH*, and *APE1* genes, which showed similar behaviors. The gefitinib and LY294002 treatments down-regulated their expression, which suggests their AKT-dependent induction. H_2_O_2_ alone did not up-regulate their expression, although a time-dependent increase in their mRNA expression was seen with gefitinib, LY294002, and PD98059 treatments. Surprisingly, the EGF treatment resulted in opposite effects on the expression of *OGG1* and *MUTYH*. Here, *OGG1* expression was reduced after 15 min of EGF treatment (p <0.01), and H_2_O_2_ addition more than reversed this effect, to result in increased *OGG1* expression over the control (p <0.001). In contrast, the *MUTYH* gene was strongly induced after 15 min of EGF treatment (p <0.001), but it was then downregulated with the addition of H_2_O_2_ (p<0.05) (**Figure 6**).

The master regulator of these antioxidant responses is the transcription factor NRF2. As shown in **Figure 6**, in this AGS cell model, induction of the antioxidant response genes *NRF2*, *HO1*, and *JUN/AP1* was independently regulated, except for inhibition of EGFR by gefitinib and of MAPK by PD98059, where *HO1* and *JUN/AP1* showed the same expression trends. Inhibition of the AKT pathway with LY294002 showed a small induction of *NRF2* gene expression, where the addition of H_2_O_2_ reduced this considerably from the control expression (p <0.01) (**Table S1**).

### Analysis for Base Excision Repair and ErbB System Correlations

**Figure 7:**
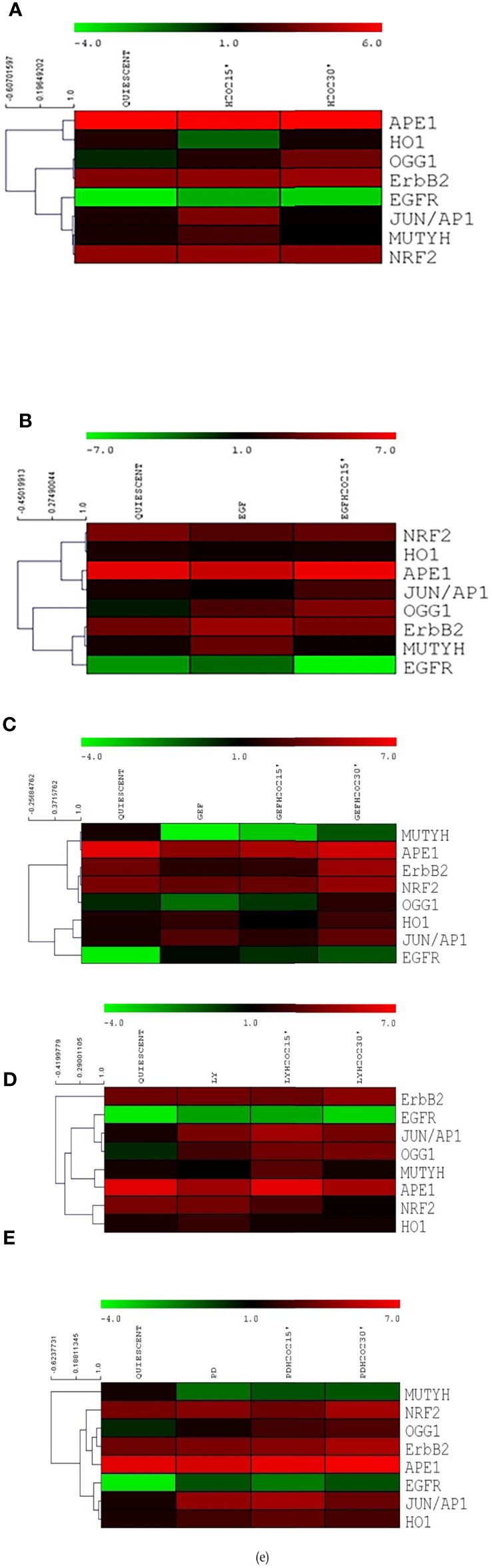
Heat maps for the components of the base excision repair and ErbB systems in AGS cells. Heat maps for serum-starved cells untreated (Quiescent) and treated with 10 mM H_2_O_2_ **(A)**, 50 ng/mL EGF **(B)**, 1 μM gefitinib (GEF) **(C)**, 25 µM LY294002 (LY) **(D)**, 50 µM PD98059 (PD) **(E)**, each **(B-E)** without and with 10 mM H_2_O_2_, for 15 and 30 min. Heat maps were constructed for expression of six genes related to base excision repair and *EGFR* and *ERBB2* (as indicated). Top, color scale: red, increase in expression; green, decrease in expression. Left, cluster dendrogram for the Spearman R correlations.

To reveal any gene expression correlations between BER and the ErbB pathways in human gastric cancer cells, we used the MeV4.0 software to define the molecular relationships among the genes assayed here, without considering that they belong to any given pathway (**Figure 7**). A strong association was detected between *ERBB2* and *OGG1* after exposure to H_2_O_2_ with the MEK1 inhibitor PD98059, with both showing symmetrical small increases after the oxidative stress. A high correlation between *ERBB2* and *MUTYH* was also seen after EGF and H_2_O_2_ co-treatments (**Figure 7B**), and among *ERBB2*, *OGG1* and *NRF2* after gefitinib and H_2_O_2_ co-treatments (**Figure 7C**). On the contrary, no correlations were seen between *ERBB2* and *MUTYH* after H_2_O_2_ and LY294002 treatment and after H_2_O_2_ and PD98059 treatment. Thus, ErbB2 appears to be involved in a strong association with some of the proteins of the BER system under conditions of acute oxidative stress. This suggests a role for ErbB2 in the modulation of DNA repair mechanisms.

## Discussion

In this study, the biological effects of short and severe H_2_O_2_ treatment on advanced gastric cancer were evaluated to highlight potential interactions with the ErbB receptor family related to tumor progression and treatment (17–19). We analyzed here the expression of the components of the ErbB and BER system in AGS gastric cancer cells, which thus resembles non-surgical traditional cancer therapies that act through the generation of ROS. Furthermore, the effects of this oxidative treatment were evaluated in combination with specific targeting agents directed against ErbB downstream signaling pathways.

Targeting a single protein has limited use for the treatment of gastric cancer because of the complex pathogenesis of this disease. Indeed, although several clinical studies have explored the effects of targeted therapies alone or in combination with chemotherapies for gastric cancer, currently only ramucirumab (anti-VEGFR2) and trastuzumab (anti-ErbB2) have been approved as gastric efficient targeted drugs (63). Therefore, the combination of targeted drugs with conventional therapies might provide new opportunities for cancer treatments.

In this study, the effects were primarily assessed for the morphology of AGS cells treated with H_2_O_2_ alone and combined with EGF pathway inhibitors (**Figure 1**) (i.e., gefitinib, LY294002, PD98509). The morphological features of these cells represent indirect signs of their functions. Other studies have shown these cells to be organized in clusters of polygon-shaped cells, with few short actin stress fibers, and no lamellipodia, and with a cobblestone-like phenotype (57). These actin filaments (in the form of stress fibers) and the thin network that forms at the edges of cells can be depolymerized by removal of serum, a phenomenon that is reversible when the cells are returned back to serum-containing medium (64).

From our observations, control untreated AGS cells grow as a heterogeneous population, with cells containing euchromatic nuclei with numerous prominent nucleoli, which is suggestive of active transcription. These different cell populations included: elongated cells, which resemble the hummingbird phenotype (**Figure 1A**, cell type H) characteristic of *H. pylori* infection (58); the presence of *H. pylori* induces the release of reactive oxygen species by host immune and epithelial cells with subsequent DNA damage to host cells (65); syncytial giant cells with multiple nuclei (**Figure 1A**, cell type S); some cells with short prolongments out of the cells (**Figure 1A**, cell type P), which suggest cell-to-cell interactions; and the majority as epithelial cells (**Figure 1A**, cell type E). In the control cells, the mild stress induced by serum starvation might be partly responsible for this cell variability. The gefitinib and PD98059 treatments did not induce any significant changes to the cell morphology. However, the proportion of epithelial-like cells was reduced, due to an increase in the rounded shape of the cells, which was mainly seen for the PD98059-treated cells. Moreover, the PI3K/AKT pathway inhibition using LY294002 induced greater stress in these cells, which led to an overall reduction in their numbers. Small, rounded cells with heterochromatic nuclei were also seen.

Oxidative stress induced by acute high-dose H_2_O_2_ treatments (10 mM for 15 min) induced major effects on all of the cell subpopulations. Small and ‘shrunken’ cells were seen for the controls, although the heterogeneity of the cell populations was preserved overall. In particular, the proportion of cells with heterochromatic nuclei increased, while there was a large reduction in the syncytial cells that are typically associated with actively growing AGS cells. Overall, these findings suggested stressor effects on these cells, showing that they were suffering (i.e., cell shrinkage), with arrest of proliferation (more heterochromatic nuclei, fewer syncytial populations), and release of microparticles. Indeed, under these oxidative stress conditions, perimembranous release of extracellular vesicles was also seen.

It has been reported that extracellular vesicles mediate intercellular communication through the release of their encapsulated materials, such as mRNAs, miRNAs, and proteins, which can then act on target cells (66). Extracellular vesicles from tumor cells can undergo cross-talk and reprograming to highly proliferative phenotype. Thus, we suggest that in these AGS cells, the oxidative stress also resulted in release of extracellular vesicles into the medium, which are more likely to mediate intercellular communications. The mRNAs and proteins released in these membrane ‘envelopes’ are believed to protect the cells from harmful stimuli, such as oxidative stress alone, and when combined with inhibitors of pro-survival pathways.

Cell proliferation was not significantly modulated after the chronic (24 h) H_2_O_2_ treatments (**Figure 2**). However, the various H_2_O_2_ co-treatments promoted strong and significant reductions in cell proliferation, which was more evident with the LY294002 treatment. This synergistic role of LY294002 and H_2_O_2_ might be explained by down-regulation of the PI3K/AKT pathway induced by the H_2_O_2_ treatment, in agreement with the protein analysis data here. It is also known that AKT confers a survival advantage to many human cancer cells (60), including gastric cancer cells (67). Thus, according to the cell morphology and cell proliferation findings here, we suggest that oxidative stress combined with PI3K/AKT pathway inhibition has a key role in the reduction of AGS cell viability.

Acquisition of migratory properties is a prerequisite for cancer progression and for invasive migration of tumor cells into the surrounding tissues. In cancer cells, acquisition of invasiveness requires a morphological alteration, which is termed epithelial–mesenchymal transition, wherein carcinoma cells lose their epithelial characteristics of cell polarity and cell–cell adhesion, and switch to a motile mesenchymal phenotype (68, 69).

In agreement with these considerations, we observed reduced cell migration after inhibition of the PI3K/AKT pathway with LY294002, even if there is no evidence of a synergistic effect with H_2_O_2_ co-treatment. In contrast, there was increased cell migration after inhibition of MEK1 with PD98059 compared with control, which thus indicates that the MAPKs constrain AGS cell motility. Interestingly, reduced migration was also seen after H_2_O_2_ treatment combined with the EGF and PD98059 treatments. This finding can be explained as reduced activation of AKT and increased activation of MAPKs, observed in protein analysis, after the H_2_O_2_ alone treatment. These data are in accordance with recent studies showing that the attenuation of PI3K/AKT signaling pathways by Ropivacaine play an inhibitory effect on the proliferation, migration and invasion of GC, thus suggesting a new therapeutic target for GC drugs (70, 71).

All these data confirmed that the AKT pathway plays a key role in conferring an aggressive phenotype to cancer cells, with the promotion of cell survival and migration and its inhibition, reproduced in gastric cancer cell line treated with LY294002, could be an important anti-cancer therapy. In the same way, a chemotherapy that increases intracellular ROS to toxic levels, reproduced with high doses of H_2_O_2_, reduced cell viability and mobility in AGS cell line. Our purpose of a combined therapy is evident in data observed with H_2_O_2_ and LY294002 co-treatment, resulting in a more noticeable reduction of cell viability. Further investigation should be performed in order to better understand the role of MAPK pathway in gastric cancer and MEK inhibition as target therapy. In this study, we observed that MAPKs inhibition with PD98059 reduced cell viability, mainly with H_2_O_2_ co-treatment, suggesting its possible anti-cancer role combined with conventional chemotherapy. However, the use of MEK1 inhibitor alone increased the ability of AGS cells to migrate, even if the cotreatment with PD98059 and H_2_O_2_ reduced migration capability compared with PD98059 alone.

Although ROS-increasing drugs are extensively used in cancer therapy, a limitation to such pro-oxidant therapies is the drug resistance that is induced in some cancer cell lines. In the gene analysis here, *ERBB2* was induced by H_2_O_2_ co-treatments with the inhibitors and by EGF treatment alone. It has already been shown that EGF can induce the *ERBB2* gene *via* ADAM12/Ets 1 molecules (72). Moreover, it has also been shown that ErbB2 overexpression in the heart can significantly decrease the levels of ROS, while the levels of glutathione peroxidase 1 and catalase are increased, along with their activities (73). This implies a defensive role for the ErbB2 receptor against oxidative stress, and thus suggests a possible induction of ErbB2 in stressed cancer cells, to defend themselves from oxidative damage. Furthermore, activation of ErbB2 leads to activation of its downstream signaling, which can then regulate apoptosis (74) and increase cell survival, which suggests a further protective role of ErbB2 in response to oxidative stress.

Indeed, increased ErbB2 expression has been associated with drug resistance in cancer cells (75). In this context, a role for oxidative stress has been evoked (76). In the human lung adenocarcinoma epithelial Calu-3 cell line, it was shown that treatment with the ErbB2-targeting antibody trastuzumab was associated with increased cellular ROS production, glutathione depletion, and decreased superoxide dismutase and catalase activities (77). Therefore, we suggest that ErbB2 induction in the AGS cells in the present study is a cellular response to oxidative stress, to protect the cells, and thus to resist H_2_O_2_-induced cell death. Accordingly, trastuzumab therapy combined with a pro-oxidant treatment should be effective through a mechanism that induces tumor cell death by-passing resistance to oxidative damage.

Base excision repair is a conserved, intracellular DNA repair system that recognizes and removes chemically modified bases, to ensure genomic integrity and prevent mutagenesis. With regard to the analysis of the BER pathway components, here we showed up-regulation of the *Ogg1* and *Mutyh* genes under the H_2_O_2_ treatment combined with these other regulators.

Previous studies by Habib et al. have also reported that the downregulation of tuberin results in a marked decrease of OGG1 expression (78, 79). In this context, AKT has been reported to phosphorylate tuberin, thus leading to its inactivation (80), and further to OGG1 down-regulation. Further studies by Staffolani et al. demonstrated that activation of EGFR/AKT/mTOR pathway induced the phosphorylation and subsequent inactivation of tuberin thus resulting in inhibition of OGG1 expression, consistent with results obtained by Habib. Therefore, the reduced expression of OGG1 determined DNA lesion accumulation, cell survival and proliferation (49).

This is in agreement with the results of the present study, which showed reduced AKT activation under H_2_O_2_ co-treatments, and up-regulation of *OGG1* gene expression. Moreover, the increased *ERBB2* gene expression under the same conditions might suggest a protective role of ErbB2 from H_2_O_2_, by up-regulation of the components of DNA repair induced by the oxidative stress.

**Figure 8:**
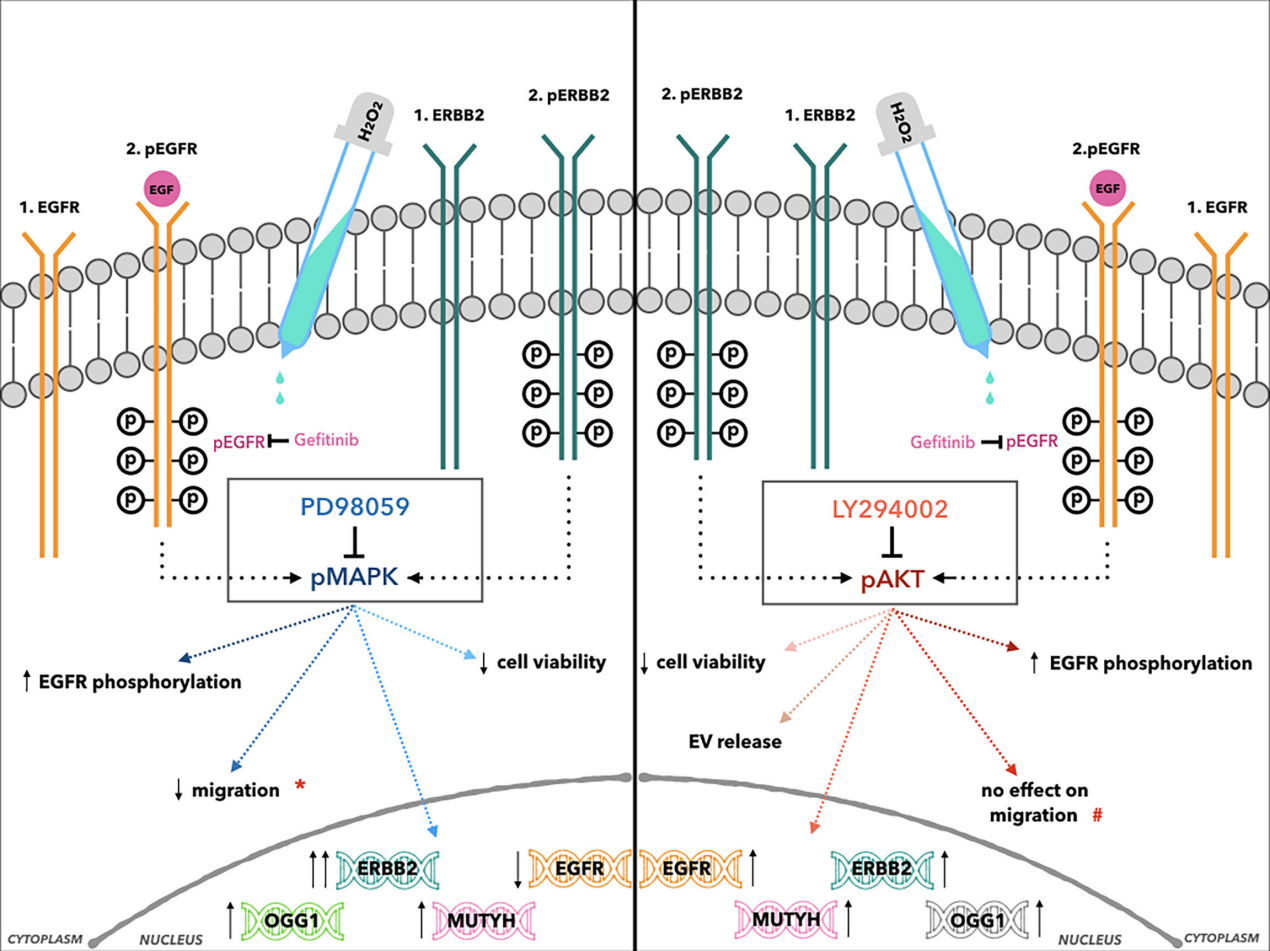
Cartoon illustration of the effects of H_2_O_2_ treatment combined with MAPK (left, central box: PD98059) and AKT inhibitors (right, central box: LY294002) in AGS cells. *Indicates the reduction of cell migration after H_2_O_2_ co-treatment compared to PD98059 alone. #Indicates the reduction of cell migration only after LY294002 alone treatment.

NRF2 modulates the expression of hundreds of genes, including the BER system antioxidant enzymes, such as *OGG1*. Thus, it also regulates the expression of genes involved in immune and inflammatory responses, carcinogenesis, and metastasis (81). The results here can also be explained by the correlations among the ErbB2 and BER system genes that were seen in the gene cluster analysis. This suggests a further negative prognostic role of ErbB2 in the induction of antioxidant genes, which can lead to the protection of cancer cells from high-dose oxidative damage induced by anticancer therapies. The effects of MAPK and AKT inhibitors in AGS cells after H_2_O_2_ co-treatment are summarized in **Figure 8**.

In conclusion, to tailor specific combinations of therapies and to decide which strategy to use, administration of a chemotherapy that increases intracellular ROS to toxic levels, might not only be dependent on the tumor type, but also on the molecular targeting therapy used. Therefore, the combination of conventional anticancer therapies with molecularly targeted therapies, such as MEK1 and PI3K inhibitors might result in side effects, i.e., ErbB2 induction, that require further studies to develop new strategies that selectively kill cancer cells and overcome drug resistance.

## Data Availability Statement

The original contributions presented in the study are included in the article/**Supplementary Materials**. Further inquiries can be directed to the corresponding author.

## Author Contributions

GM, LS, and MCDM contributed to study design, data curation and wrote the manuscript. MCDM, LS, and CM performed cell cultures, protein and gene expression analysis. GMA validated the replication of experiments on gene expression analysis. LC carried out cell morphology analysis. GM, RC, and RM critically reviewed the manuscript. GM designed and coordinated the study and drafted the manuscript. All authors contributed to data analysis, drafting or revising the article, gave final approval of the version to be published, and agree to be accountable for all aspects of the work.

## Funding

This study was supported by the “G. d’Annunzio” University of Chieti-Pescara (Fondi di Ateneo per la Ricerca-F.A.R.) grant to GMA, RM, and GMA.

## Conflict of Interest

The authors declare that the research was conducted in the absence of any commercial or financial relationships that could be construed as a potential conflict of interest.

The reviewer ID’A declared a shared affiliation, with no collaboration, with the authors, to the handling editor at the time of review.

## Publisher’s Note

All claims expressed in this article are solely those of the authors and do not necessarily represent those of their affiliated organizations, or those of the publisher, the editors and the reviewers. Any product that may be evaluated in this article, or claim that may be made by its manufacturer, is not guaranteed or endorsed by the publisher.
