# Supplementary material for: Effects of H2O2 Treatment Combined With PI3K Inhibitor and MEK Inhibitor in AGS Cells: Oxidative Stress Outcomes in a Model of Gastric Cancer
Source: Front Oncol. 2022 Mar 16;12:860760. doi: 10.3389/fonc.2022.860760 (PMC8966616; doi:10.3389/fonc.2022.860760)
Supplement: Supplementary file 1 [file DataSheet_1.docx]

Supplementary Material

# Supplementary Figures and Tables

##
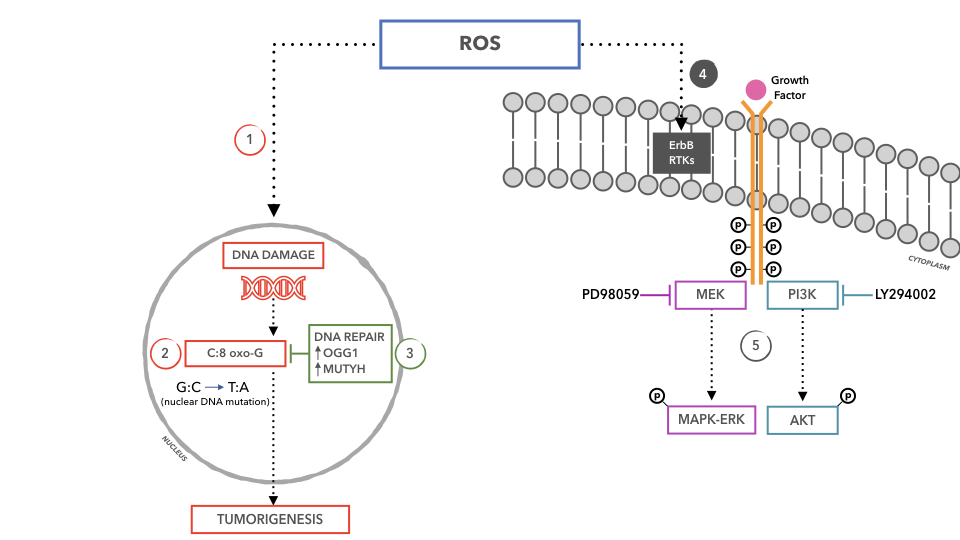
Figures S1

**Schematic representation of BER and ErbB systems. (1)** Elevated ROS cause nuclear DNA damage. **(2)** High levels of ROS oxidize guanine in DNA, resulting in the formation of 8-oxo-G base pairs through oxidation of G, which leads to G→T transversion mutation. **(3)** This mutation can be recognized by OGG1, which excises the 8-oxo-G. **(4)** ROS have also been shown to directly activate ErbB RTKs. **(5)** The activation of ErbB receptors results in transphosphorylation of the ErbB dimer partner, stimulating intracellular pathways including MEK/ERK and PI3K/AKT.

**Table S1. O**[**ne-way ANOVA**](https://www.statology.org/one-way-anova/) **analysis of gene expression results.**

| **Gene** | **treatment** | **SS** | **df** | **MS** | **F** | ***p-value*** |
| --- | --- | --- | --- | --- | --- | --- |
| ***APE-1*** | GEF; GEF + H_2_O_2_ 15’; GEF + H_2_O_2_ 30’ | 3.058 | 2 | 1.529 | 21.959 | 0.002 |
|  | LY; LY + H_2_O_2_ 15’; LY + H_2_O_2_ 30’ | 5.283 | 2 | 2.641 | 591.336 | <0.001 |
|  | PD; PD + H_2_O_2_ 15’; PD + H_2_O_2_ 30’ | 0.973 | 2 | 0.486 | 19.327 | 0.002 |
| ***EGFR*** | GEF; GEF + H_2_O_2_ 15’; GEF + H_2_O_2_ 30’ | 3.198 | 2 | 1.599 | 193.44 | <0.001 |
|  | LY; LY + H_2_O_2_ 15’; LY + H_2_O_2_ 30’ | 1.882 | 2 | 0.941 | 2.145 | 0.198 |
|  | PD; PD + H_2_O_2_ 15’; PD + H_2_O_2_ 30’ | 0.31 | 2 | 0.155 | 2.683 | 0.136 |
| ***ErbB2*** | GEF; GEF + H_2_O_2_ 15’; GEF + H_2_O_2_ 30’ | 13.92 | 2 | 6.96 | 743.071 | <0.001 |
|  | LY; LY + H_2_O_2_ 15’; LY + H_2_O_2_ 30’ | 1.037 | 2 | 0.519 | 273 | <0.001 |
|  | PD; PD + H_2_O_2_ 15’; PD + H_2_O_2_ 30’ | 2.225 | 2 | 1.113 | 16.517 | 0.004 |
| ***NRF2*** | GEF; GEF + H_2_O_2_ 15’; GEF + H_2_O_2_ 30’ | 2.467 | 2 | 1.233 | 15.996 | 0.004 |
|  | LY; LY + H_2_O_2_ 15’; LY + H_2_O_2_ 30’ | 9.5 | 2 | 4.75 | 2,638.89 | <0.001 |
|  | PD; PD + H_2_O_2_ 15’; PD + H_2_O_2_ 30’ | 2.173 | 2 | 1.087 | 21.72 | 0.002 |
| ***OGG1*** | GEF; GEF + H_2_O_2_ 15’; GEF + H_2_O_2_ 30’ | 14.907 | 2 | 7.453 | 85.051 | <0.001 |
|  | LY; LY + H_2_O_2_ 15’; LY + H_2_O_2_ 30’ | 3.567 | 2 | 1.784 | 131.793 | <0.001 |
|  | PD; PD + H_2_O_2_ 15’; PD + H_2_O_2_ 30’ | 2.998 | 2 | 1.499 | 307.993 | <0.001 |
| ***MUTYH*** | GEF; GEF + H_2_O_2_ 15’; GEF + H_2_O_2_ 30’ | 15.493 | 2 | 7.746 | 358.072 | <0.001 |
|  | LY; LY + H_2_O_2_ 15’; LY + H_2_O_2_ 30’ | 13.893 | 2 | 6.946 | 21.64 | 0.002 |
|  | PD; PD + H_2_O_2_ 15’; PD + H_2_O_2_ 30’ | 0.538 | 2 | 0.269 | 6.413 | 0.032 |
| ***HO-1*** | GEF; GEF + H_2_O_2_ 15’; GEF + H_2_O_2_ 30’ | 3.095 | 2 | 1.548 | 20.176 | 0.002 |
|  | LY; LY + H_2_O_2_ 15’; LY + H_2_O_2_ 30’ | 1.516 | 2 | 0.758 | 20.198 | 0.002 |
|  | PD; PD + H_2_O_2_ 15’; PD + H_2_O_2_ 30’ | 0.702 | 2 | 0.351 | 4.901 | 0.055 |
| ***JUN/AP1*** | GEF; GEF + H_2_O_2_ 15’; GEF + H_2_O_2_ 30’ | 2.831 | 2 | 1.416 | 128.7 | <0.001 |
|  | LY; LY + H_2_O_2_ 15’; LY + H_2_O_2_ 30’ | 1.834 | 2 | 0.917 | 1,448.21 | <0.001 |
|  | PD; PD + H_2_O_2_ 15’; PD + H_2_O_2_ 30’ | 3.85 | 2 | 1.925 | 1,650.17 | <0.001 |

O[ne-way ANOVA](https://www.statology.org/one-way-anova/) was employed to determine, for each single gene, statistically significant difference between the means of the treatments presenting three points (GEF; GEF + H_2_O_2_ 15’; GEF + H_2_O_2_ 30’, LY; LY + H_2_O_2_ 15’; LY + H_2_O_2_ 30’, PD; PD + H_2_O_2_ 15’; PD + H_2_O_2_ 30’). Statistical significance was accepted at p <0.05. GEF: gefitinib; LY: LY294002; PD: PD98059.

GEF: Gefitinib; LY: LY294002; PD: PD98059.
